# Evaluation of the Wearable Eye Tracker Pupil Labs Neon for Gaze Fixation Analysis in Screen-Based Fixation Tasks: Comparison with Tobii Pro Fusion

**DOI:** 10.3390/jemr19050098

**Published:** 2026-09-05

**Authors:** Madara Alecka, Evita Serpa, Viktorija Arhipenko, Linda Krauze, Ilze Ceple

**Affiliations:** Department of Optometry and Vision Science, Faculty of Science and Technology, University of Latvia, Jelgavas Street 1, LV-1004 Riga, Latvia

**Keywords:** video-oculography, eye-tracking accuracy, fixation stability, wearable eye tracker, gaze analysis

## Abstract

Wearable eye-tracking devices are primarily used for eye movement analysis in real-world environments. Owing to their increasing availability and ability to provide measurements with high ecological validity, it is important to determine whether they can also be applied to gaze fixation analysis involving screen-based stimuli. This study evaluated the performance of the wearable eye tracker Pupil Labs Neon for gaze fixation analysis under controlled laboratory conditions by comparing it with the screen-based Tobii Pro Fusion. A total of 46 participants were enrolled in the study. Eye-tracker accuracy was assessed across different gaze directions, whereas fixation stability was evaluated exclusively during central fixation. Tobii Pro Fusion demonstrated significantly higher overall accuracy compared to Pupil Labs Neon. Notably, the accuracy of both devices was affected by stimulus background. Furthermore, the Tobii Pro Fusion showed significantly better accuracy at central positions than at peripheral gaze positions; in contrast the Pupil Labs Neon exhibited no significant variations in relation to stimulus eccentricity. Finally, no significant differences in fixation stability during central fixation were observed between the two devices. Considering its significantly higher overall accuracy, Tobii Pro Fusion appears better suited for gaze fixation analysis in screen-based tasks, especially within the central positions, whereas Pupil Labs Neon may provide more stable data quality across different gaze positions.

## 1. Introduction

Video-oculography is a widely applied non-invasive method for tracking and recording eye movements. It utilizes multiple built-in video cameras and one or more infrared light sources to enable the localization of reflections from the optical structures of the eye and the subsequent analysis, tracking, and recording of eye movement trajectories [1]. This method allows for the precise determination of gaze direction and distinguishes whether movement is caused by eye or head motion, based on changes in the relative position between the corneal reflection and the center of the pupil. With additional structural analysis, such as iris texture and scleral blood vessel patterns, the device is capable of recording not only horizontal and vertical eye movements but also torsional eye movements. These systems can also provide information about pupil size and position, as well as characteristics of fixations and saccadic velocity. Video-oculographs are capable of ensuring high accuracy in eye movement recording, thereby contributing both to the diagnosis of various neurological disorders and to advancements in psychophysiology and cognitive neuroscience [1,2].

Eye movement recording devices can be either stationary or mobile. Stationary eye-tracking systems are typically used in a laboratory setting, as they require a fixed position relative to the object being studied. These systems use one or more built-in cameras positioned in a fixed relation to the subject’s eyes and head to obtain precise eye movement data [3]. The sampling frequencies of stationary systems are generally higher than those of other types of devices, resulting in more precise characterization of eye movements [4,5]. However, data collection may be more challenging and less accurate for participants with refractive errors, as reflections from eyeglasses or contact lenses may interfere with eye tracking [6].

Mobile or head-mounted eye-tracking devices, often designed in the form of glasses, provide broader opportunities for eye movement research. These devices allow for the study of eye movements in real-world, dynamic environments outside of a laboratory setting. This is possible because the devices include multiple cameras directed at both the eyes and the surrounding environment, enabling eye movement data to be recorded within the device’s coordinate system, and because they can operate while connected to a smart device or a laptop. Devices of this type, such as Pupil Labs Core, Pupil Labs Neon, and Tobii Pro Glasses 3, offer the possibility of incorporating individualized spherical corrective lenses using the device’s frame [3,7]. The main disadvantage of such devices is that the eyes must remain open throughout the entire measurement, meaning that even brief blinks can interfere with accurate data acquisition [8].

Device parameters that play a role in selecting an eye-tracking system for studying gaze-related parameters include the device’s accuracy, which is defined as the angular deviation between the gaze position recorded by the device and the actual target position [9]. Typically, device manuals report an accuracy of approximately 0.5°, but it has been found that under real-world conditions, accuracy often varies between 1° and 2° [10,11,12]. The obtained accuracy is also influenced by the type of device used, as stationary eye-tracking systems generally demonstrate higher accuracy than mobile systems [11]. In addition to accuracy, device precision is also often evaluated. This is characterized by the standard deviation or RMS noise level, representing the dispersion of gaze points during fixation. The precision of eye-tracking devices typically ranges from 0.05° to 0.1° [9,13]. Both parameters are essential for accurately characterizing fixation stability, which is derived from fixation eye movements such as microsaccades, drift, and tremor, including their frequency and amplitude.

As critical indicators of underlying visual function, fixation characteristics serve as valuable biomarkers for assessing both ocular health and cognitive processes [14,15,16]. It is commonly described using the Bivariate Contour Ellipse Area (BCEA), which represents the area of an ellipse calculated from all recorded fixation points along the horizontal and vertical axes. This value is expressed in degrees as a two-dimensional area. Fixation stability can also be characterized by the proportion of gaze position samples located within a defined area during fixation. If more than 75% of gaze points fall within a region with a diameter of less than 2°, fixation is considered stable [17]. In contrast, greater dispersion of fixation points and a higher number of microsaccades during fixation indicate less stable fixation [18].

Since the design principles of eye-tracking devices vary between manufacturers, a wide range of such devices are available on the market. Although all eye-tracking devices share the common goal of recording eye movements, the methods used to achieve this differ. As a result, some devices are more suitable for laboratory use, while others are better adapted for real-world environments [19]. Although wearable eye trackers are primarily considered suitable for studies conducted in real-world environments, the number of studies employing such devices has been increasing [20]. Given the increasing number of studies employing wearable eye trackers and the high level of ecological validity provided by wearable eye-tracking systems, the present study investigates their applicability to gaze fixation analysis tasks that are traditionally performed under laboratory conditions. Specifically, this study aims to evaluate the performance of the wearable eye-tracking device Pupil Labs Neon in gaze fixation stability analysis and to assess its accuracy across different gaze directions under controlled stimulus presentation conditions in comparison with the screen-based eye-tracking device Tobii Pro Fusion.

## 2. Method

### 2.1. Participants

Two data analyses were performed in this study to evaluate different outcome measures. The analysis of eye-trackers accuracy included 46 participants (22 ± 2 years), while the fixation stability analysis included 32 participants (22 ± 2 years). Participants were required to have a binocular visual acuity of at least 0.6 (decimal units) at a viewing distance of 40 cm, either uncorrected or corrected with contact lenses, and binocular single vision. Additionally, participants were required to be free of general health or ocular conditions, such as Huntington’s disease, myasthenia, nystagmus, or Graves’ disease, which could affect the obtained fixation stability measurements. Visual acuity was assessed using a near Snellen chart, and the presence of binocular single vision was determined using the TNO test.

The study was conducted in accordance with the Declaration of Helsinki. It was approved by the Research Ethics Committee for Optometry and Vision Science, Faculty of Sciences and Technology, University of Latvia, on 29 January 2025 (Approval No. 19-121/10). Written informed consent for data processing was obtained from all participants, who enrolled in the study on a voluntary basis.

### 2.2. Equipment and Tools

During the study, two video-oculographs with different design principles were compared: the stationary screen-based Tobii Pro Fusion (Tobii AB, Stockholm, Sweden) and the wearable Pupil Labs Neon (Pupil Labs, Berlin, Germany).

To record gaze direction and fixation parameters, the Tobii Pro Fusion eye-tracking system utilizes video-based pupil-center corneal reflection (PCCR) technology integrated within a three-dimensional eye model. The system illuminates the eye with near-infrared light, capturing two primary features: the center of the pupil and the corneal reflex. The spatial vector between the pupil center and the corneal reflex is continuously calculated to determine precise gaze direction. Because minor head movements displace both features uniformly whereas eye rotations shift only the pupil center relative to the corneal reflex, this vector tracking effectively isolates true gaze behavior from head movements. Alongside these spatial coordinates, the system concurrently measures pupil diameter, allowing for simultaneous evaluation of spatial gaze metrics and pupil-related dynamics. To ensure higher data quality, the device applies two eye-tracking cameras with two pupil detection modes (dark and bright pupil). In the current study, the dark pupil mode was applied. The sampling frequency of the video-oculograph during the study was 250 Hz.

**Figure 1:**
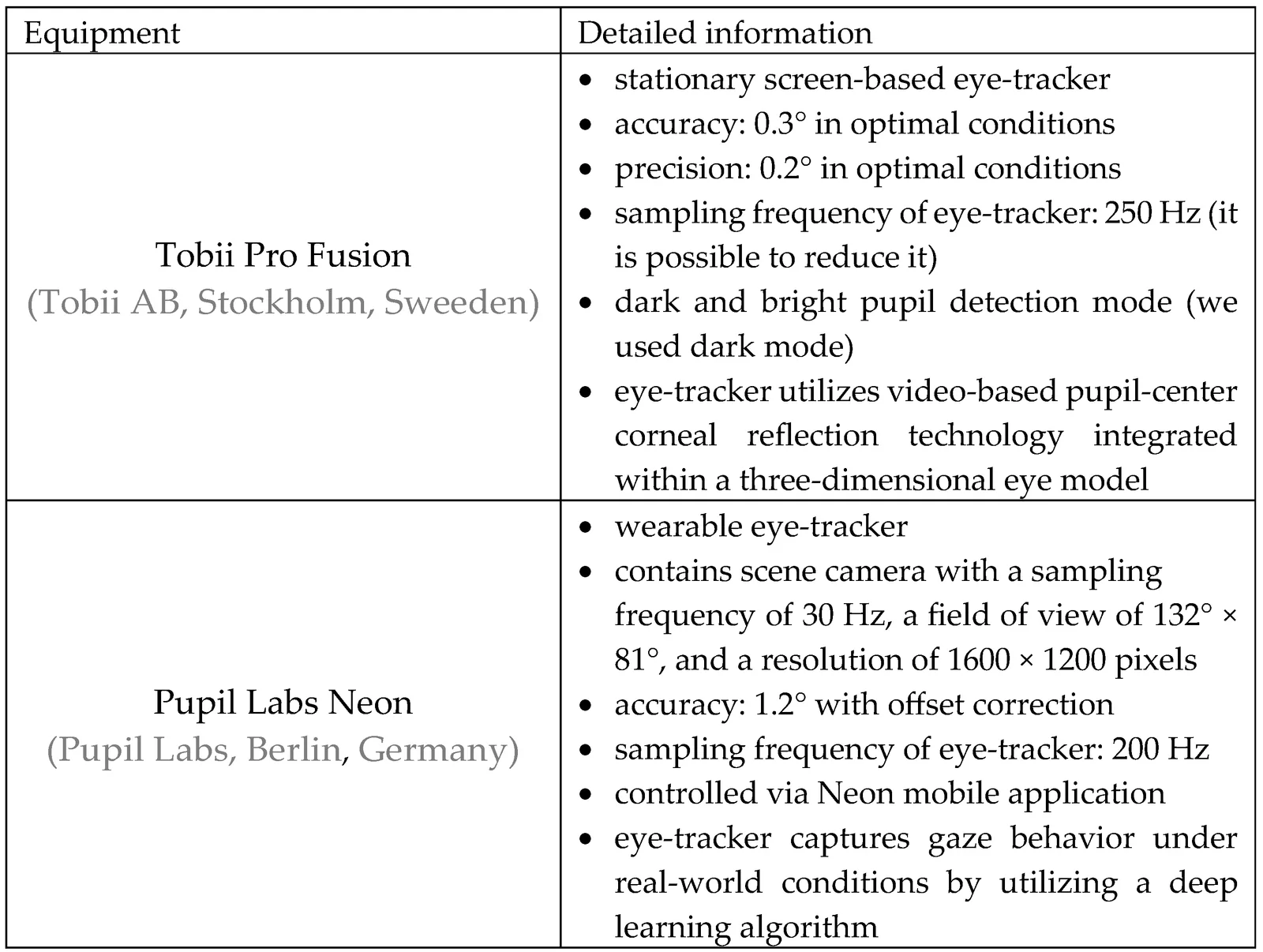
Technical details of equipment used in study.

The Pupil Labs Neon eye-tracker captures gaze behavior under real-world conditions by utilizing a deep learning algorithm (NeonNet) to estimate 3D gaze direction directly from eye video data. This machine-learning approach directly maps gaze coordinates, allowing for the quantification of fixations and fixation-related eye movements in dynamic environments. The device consists of a central Neon module, which includes a scene camera with a sampling frequency of 30 Hz, a field of view of 132° × 81°, and a resolution of 1600 × 1200 pixels, as well as two infrared eye-tracking cameras operating at 200 Hz. Additionally, the system is equipped with a stereo microphone and an inertial measurement unit (IMU) that records linear acceleration, angular velocity, and magnetic field parameters at 110 Hz. The integration of the IMU enables precise tracking of head movements and the spatial orientation of the device, which is particularly important for gaze analysis in dynamic environments. The video-oculograph is controlled via the Neon mobile application developed by Pupil Labs. This application allows for real-time evaluation of gaze direction, improvement of measurement accuracy by specifying the individual interpupillary distance, and manual gaze calibration. Recorded data are stored and accessed through the Pupil Cloud platform, which uses deep machine learning algorithms trained on diverse datasets to ensure robust gaze estimation regardless of individual anatomical differences or varying lighting conditions (see Figure 1) [21].

### 2.3. Display

A DELL IPS-type LCD widescreen monitor (model: P2419H) was used to present fixation stimuli. The screen dimensions were 52.70 × 29.64 cm, with a resolution of 1920 × 1080 pixels.

### 2.4. Fixation Stimulus

**Figure 2:**
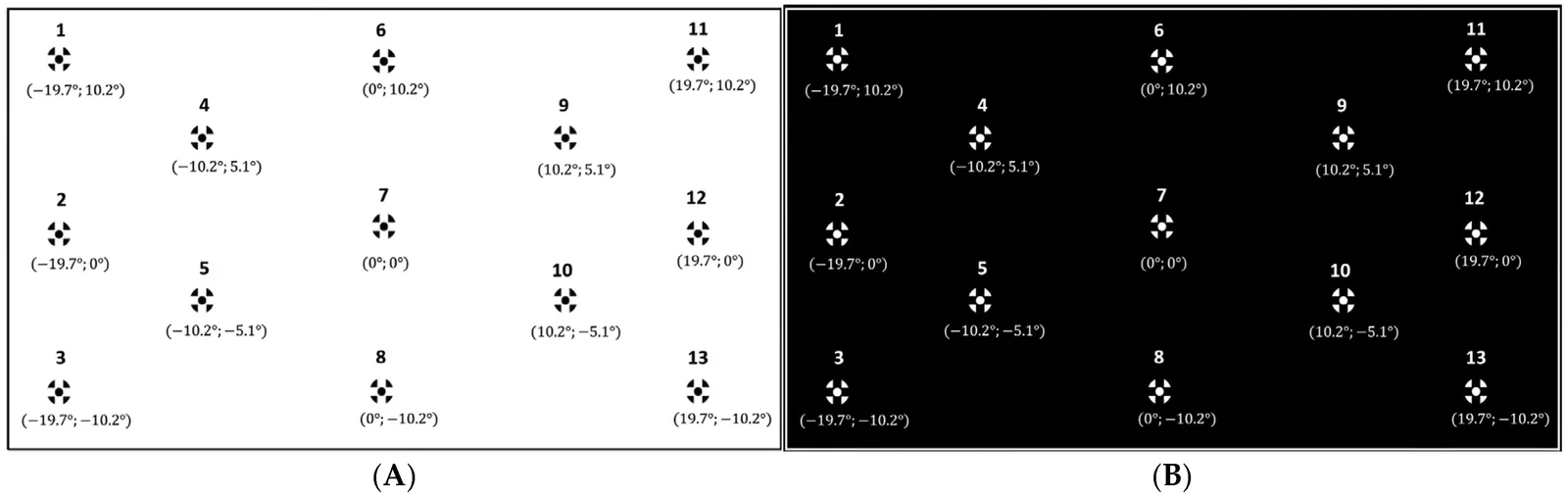
Arrangement of fixation stimuli on the monitor screen and their positional numbering (**A**) on white screen and (**B**) on black screen.

In total, two types of stimuli were applied. Stimulus one consisted of a 0.6° black circle with a white cross and a 0.2° black fixation point at its center. This stimulus was presented on a white background (RGB 255, 255, 255) at 13 different positions on the screen (see Figure 2). The second fixation stimulus consisted of a 0.6° white circle containing a black cross, with a 0.2° white fixation point at its center. This stimulus was presented on a black background (RGB 0, 0, 0) at 13 different positions on the screen [18]. The horizontal distance between fixation stimuli was 23.3 cm (19.7°), the vertical distance was 11.6 cm (10.1°), and the diagonal distance was 13 cm (11.3°).

### 2.5. Experimental Design and Procedure

The experiment involved two outcome measures: eye-tracker accuracy and fixation stability. Accuracy was compared between the Pupil Labs Neon and Tobii Pro Fusion eye trackers across different fixation target positions on both white and the black backgrounds, while fixation stability was evaluated only within the central fixation when the stimuli appeared on a white background.

**Figure 3:**
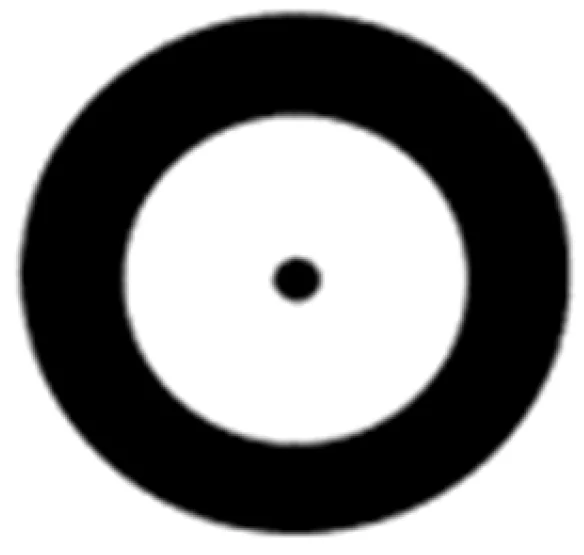
Calibration stimulus of the devices.

At the beginning of the experiment, participants received information about the study, provided written informed consent, and were given the opportunity to ask questions. Demographic information and general and ocular history were collected. Visual acuity and the presence of binocular single vision were assessed to confirm that participants met the inclusion criteria. Interpupillary distance was also measured as it is required for Pupil Labs Neon recordings. During measurements with each device, the participant’s head was stabilized using a forehead and chin rest at a distance of 65 cm from the monitor. The testing order of the eye-tracking devices was randomized to counter potential order effects. Before the measurements, each device was calibrated for the specific participant. The Tobii Pro Fusion eye tracker was calibrated using a 5-point calibration and 4-point validation under binocular conditions. If the mean gaze direction error exceeded 0.5° or the dispersion exceeded 0.2–0.3°, the calibration and validation procedures were repeated. In contrast, the Pupil Labs Neon eye tracker applied a one-point calibration method, during which the examiner specified the participant’s interpupillary distance and gaze offset using a calibration point in the Neon application. For device calibration, a black circular stimulus containing a smaller white circle with a central black fixation point was used. The calibration stimulus was presented on a white background (RGB 255, 255, 255), regardless of the color of the stimulus used in the subsequent task (see Figure 3).

**Figure 4:**
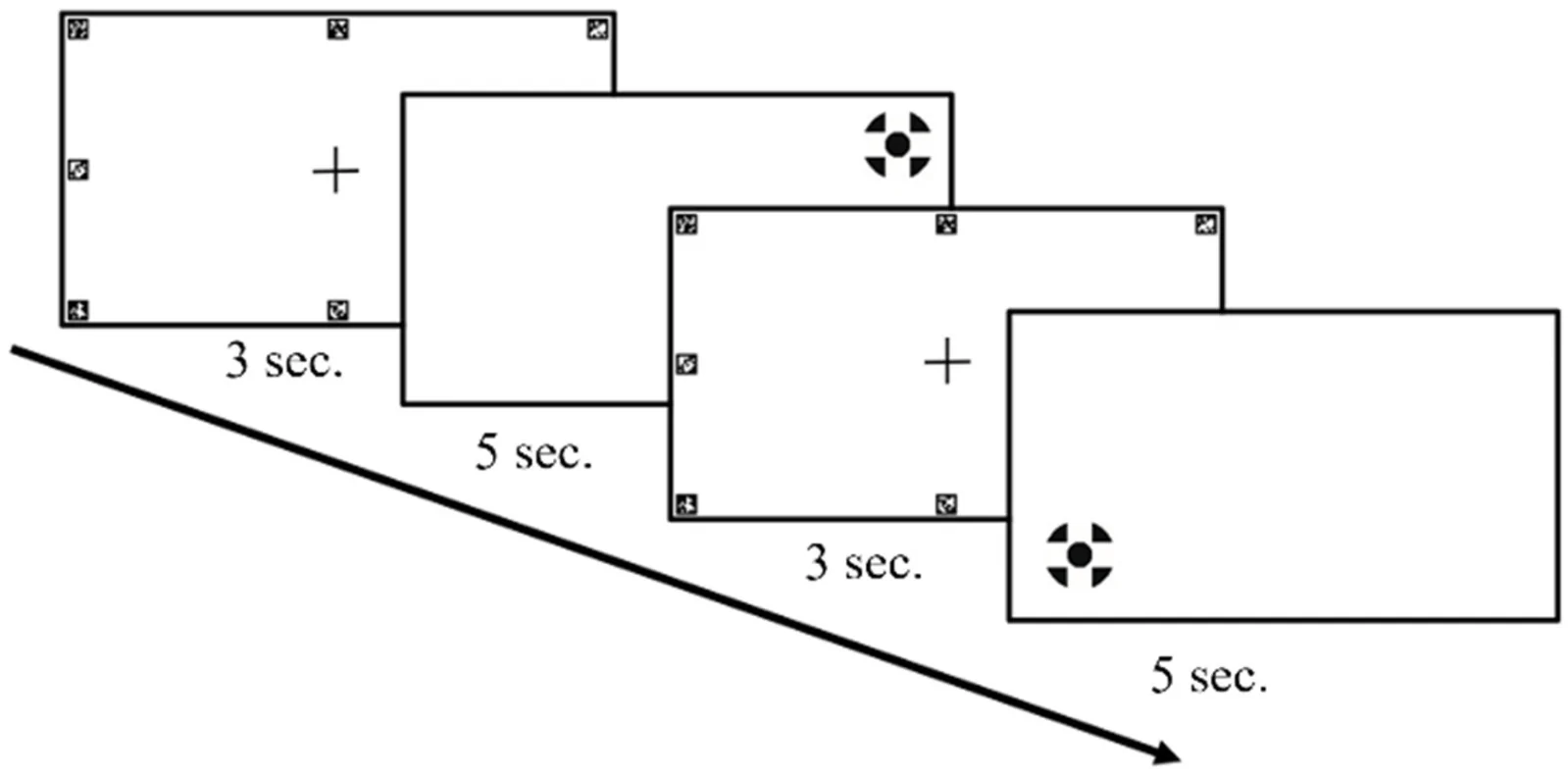
Task procedure.

Calibration was followed by the task execution, during which 13 fixation stimuli were presented on the screen at different positions in a randomized order, each for 5 s. Between each stimulus, a 1.38° fixation cross was displayed in the center of the screen for 3 s to ensure that the starting position for each fixation stimulus was the same (see Figure 4). Measurements on white and black backgrounds were performed in a randomized order. The participant’s task was to fixate both on the fixation stimulus and on the central fixation cross.

### 2.6. Data Analysis

Fixation accuracy was analysed based on gaze direction coordinates (raw data) recorded by both eye-tracking devices on the monitor screen. Only data corresponding to fixation on the given stimulus were included in the analysis; data related to blinks and saccades between stimuli were excluded. For the Pupil Labs Neon device, gaze direction coordinates on the screen were obtained using the Reference Image Mapper tool available in the Pupil Cloud platform as it determines gaze coordinates relative to a defined static image or object rather than a video frame. To determine the actual accuracy of the eye-tracking devices, the mean *x* and *y* gaze coordinates were calculated from fixation data corresponding to the presented stimulus.

#### 2.6.1. Computation of Actual Accuracy of the Eye-Tracking Devices

Accuracy was defined as the Euclidean distance between the actual position of the fixation stimulus and the recorded gaze coordinates on the screen.

**Figure 5:**
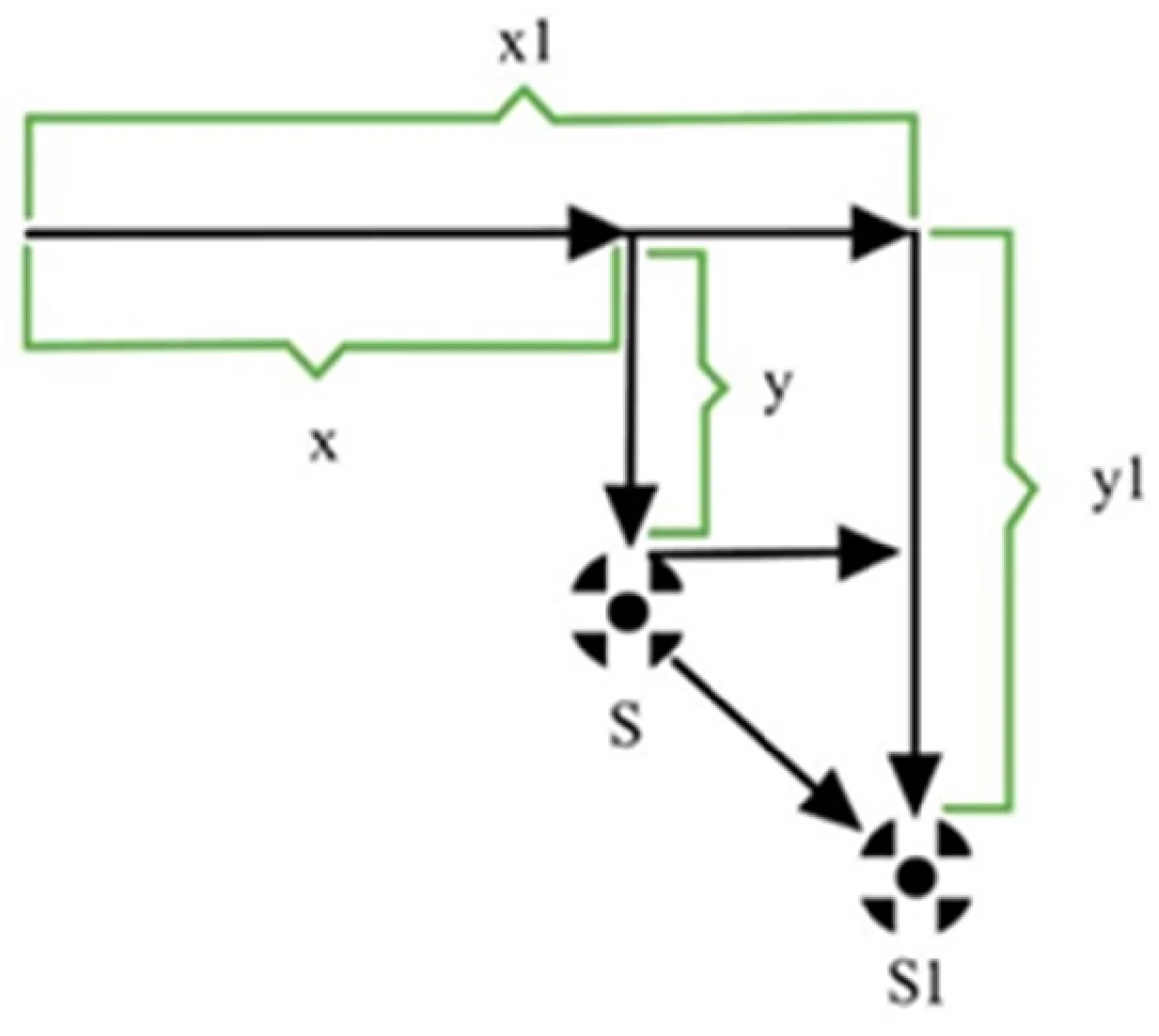
Accuracy calculation.

Device accuracy was calculated using the formula:

$$SS1\;=\;\surd (x1-x{)}^{2}\;+\;(y1-y{)}^{2},$$

where *S* is the fixation object, *S*1 is the fixation point estimated by the device, *x*1 and *y*1 are the recorded fixation coordinates, and *x* and *y* are the true coordinates of the fixation stimulus (see Figure 5). Initially, device accuracy was calculated in pixels and later converted to centimeters. The average accuracy of participants’ eye movements at each stimulus point was expressed in degrees.

#### 2.6.2. Computation of BCEA Values

The I2MC algorithm implemented in MATLAB was used to calculate fixation stability (BCEA) values throughout the task. This algorithm is suitable for datasets with significant noise or frequent data loss [22].

The following formula was used to calculate fixation stability values:

$$BCEA\;=\;2k\pi \sigma H\sigma V(1-\rho^{2}{)}^{1/2},$$

where *k* is 1.14, *σH* is the standard deviation of fixations along the horizontal meridian, *σV* is the standard deviation of fixations along the vertical meridian, and *ρ* is the Pearson correlation coefficient between the two meridians [23]. The first 0.5 s of recorded data were excluded from the BCEA calculation to avoid inaccuracies that could arise if the participant had not yet fixated on the stimulus at the beginning.

### 2.7. Data Processing

For data processing and statistical analysis, the following software were applied: MATLAB R2020a, Microsoft Excel (Microsoft 365, version 2412), RStudio (R version 4.4.1), IBM SPSS Statistics 29.0, Tobii Pro Lab, and the Pupil Cloud platform. Non-parametric tests were applied to all data due to the small sample size and the violation of normality assumptions (*p* < 0.05). Different statistical tests were used for each experiment. The Shapiro–Wilk test was used on raw data to assess normality as related samples were compared. The Wilcoxon signed-rank test was used to compare the accuracy of the two eye-tracking devices, the obtained fixation stability values between the devices and across different stimulus positions, as well as the differences in device accuracy between black and white backgrounds. The non-parametric Friedman test was used to evaluate whether the position of the stimulus on the screen affected gaze accuracy. The Dunn–Bonferroni post hoc test was applied to determine which specific fixation stimulus positions showed statistically significant differences.

## 3. Results

### 3.1. Accuracy

#### 3.1.1. Accuracy of Eye-Tracking Devices

**Figure 6:**
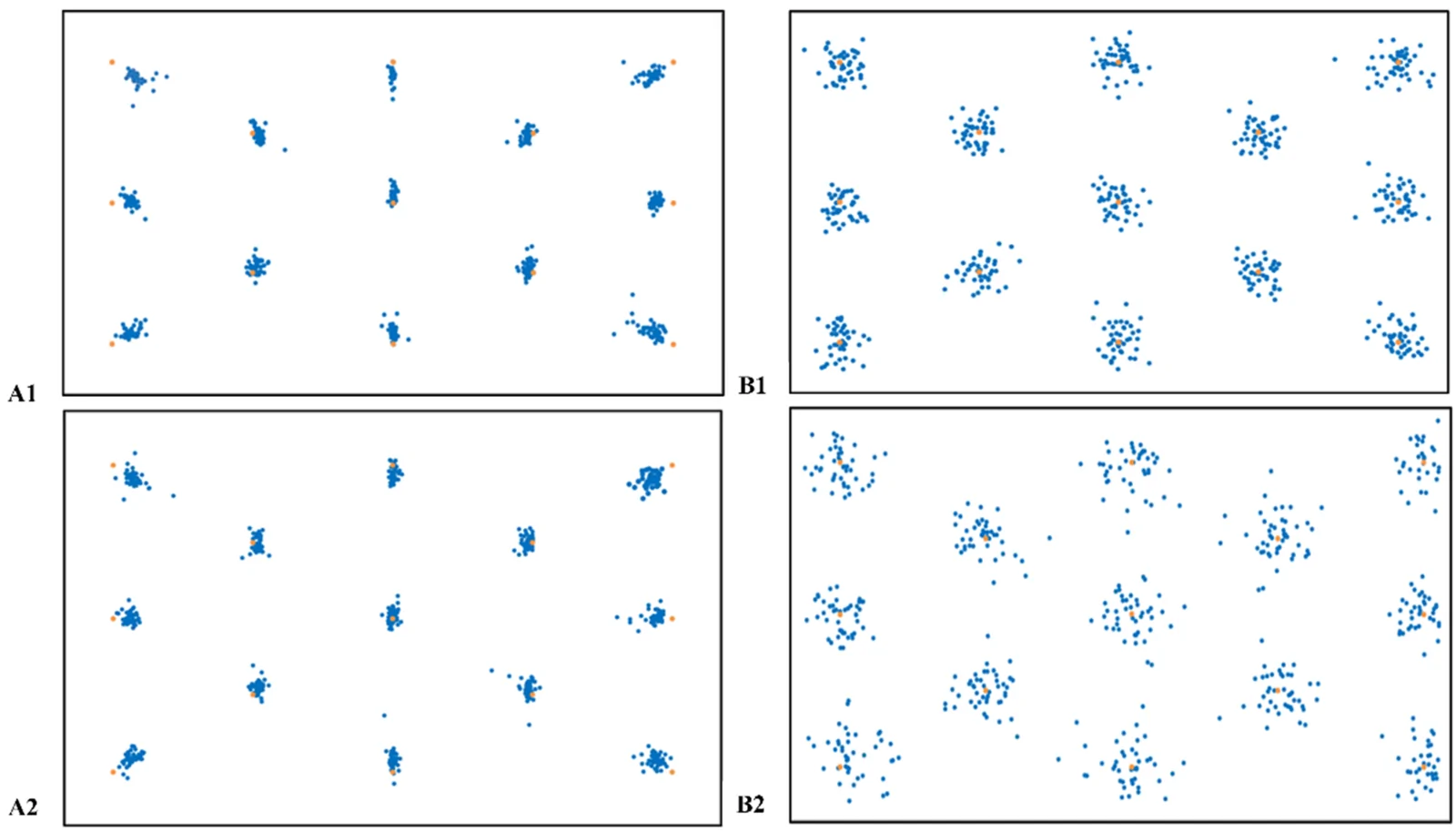
Locations of recorded points (blue dots) and actual stimuli (orange dots) for all participants. (**A1**) Results obtained with the Tobii Pro Fusion device on a white background, (**B1**) results obtained with the Pupil Labs Neon device on a white background, (**A2**) results obtained with the Tobii Pro Fusion device on a black background, (**B2**) results obtained with the Pupil Labs Neon device on a black background.

By calculating the mean deviation between 13 gaze fixation points on both white and black backgrounds, it was found that the average eye-tracking accuracy of the Tobii Pro Fusion video-oculograph is higher (1.23° ± 0.79°) than that of the Pupil Labs Neon video-oculograph (1.41° ± 0.92°). The Wilcoxon signed-rank test indicated that there is a statistically significant difference between the accuracies of the two devices (Z = −4.29; *p* < 0.001). When examining each background color separately, on a white background, the Tobii Pro Fusion video-oculograph demonstrated a mean accuracy of 1.35° ± 0.85°, while the Pupil Labs Neon video-oculograph showed a mean accuracy of 1.20° ± 0.72°. A statistically significant difference between device accuracies was observed (Z = −3.43; *p* < 0.001). On a black background, the Tobii Pro Fusion video-oculograph showed an accuracy of 1.10° ± 0.70°, whereas the Pupil Labs Neon video-oculograph showed an accuracy of 1.63° ± 1.05°. The Wilcoxon signed-rank test again indicated a statistically significant difference between the accuracies of the two devices (Z = −5.27; *p* < 0.001) (see Figure 6).

#### 3.1.2. Effect of Gaze Direction on Eye-Tracking Device Accuracy

**Figure 7:**
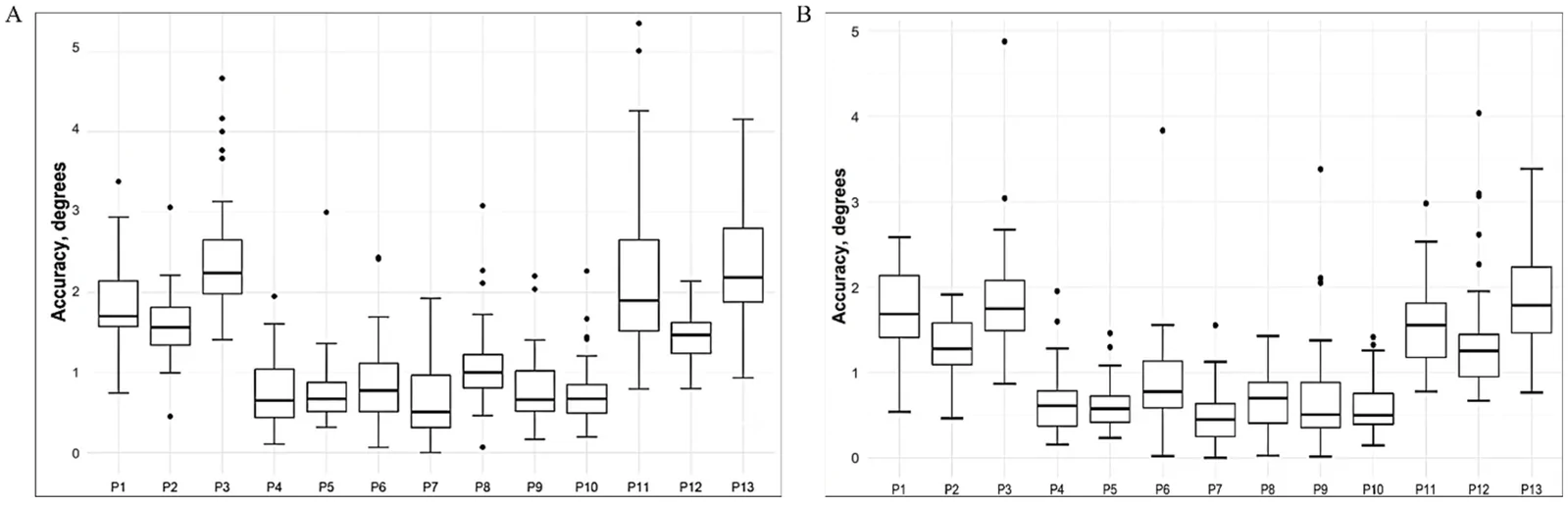
Data deviation from the stimulus location obtained with Tobii Pro Fusion on a white background (**A**) and on a black background (**B**). The horizontal line within each box represents the median value.

Further analysis of how eye-tracking device accuracy varies depending on the position of the gaze fixation stimulus on the screen showed that the accuracy of the Tobii Pro Fusion device depends on the position of the fixation stimulus on both white (χ^2^ = 362.88; *p* < 0.001) and black (χ^2^ = 344.61; *p* < 0.001) backgrounds, as determined by the Friedman test (see Figure 7).

**Figure 8:**
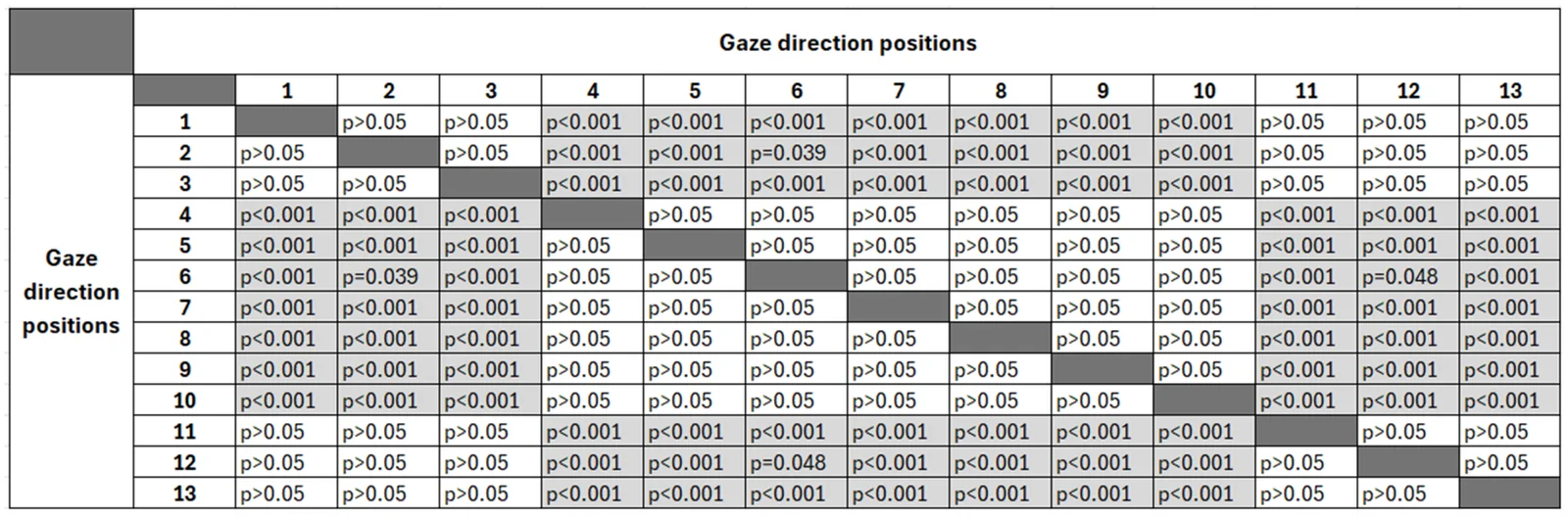
Dunn–Bonferroni post hoc test results for Tobii Pro Fusion with stimuli on a black background. Gray shading indicates statistical significance (*p* < 0.05).

**Figure 9:**
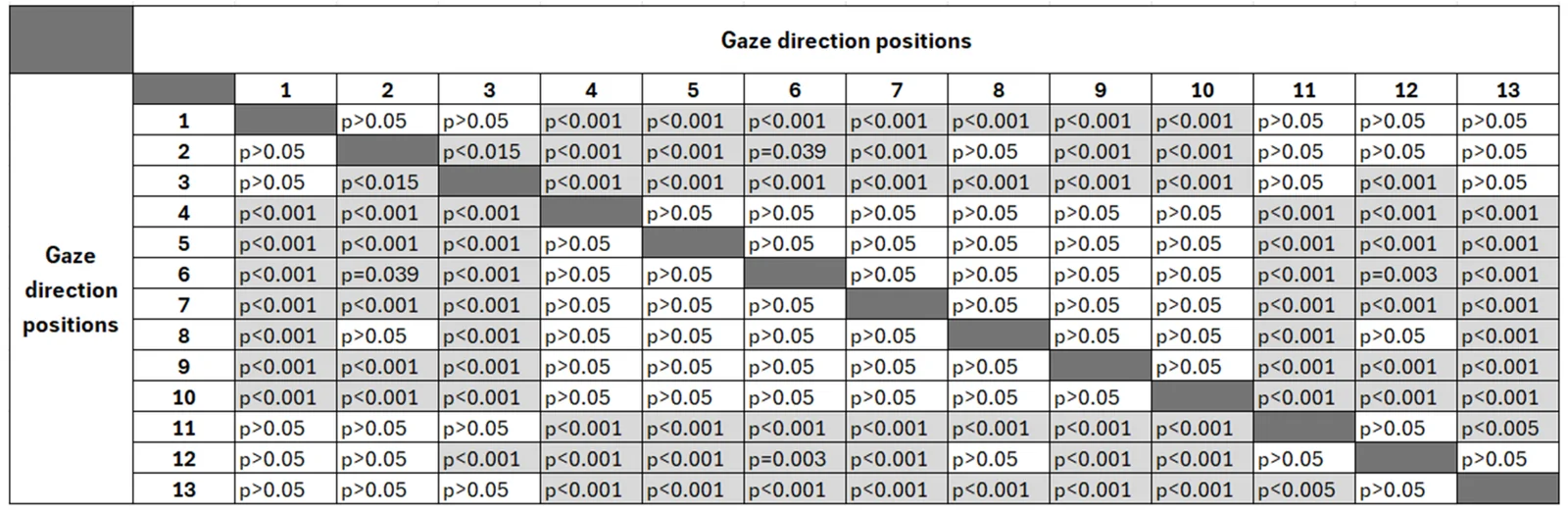
Dunn–Bonferroni post hoc test results for Tobii Pro Fusion with stimuli on a white background. Gray shading indicates statistical significance (*p* < 0.05).

The Dunn–Bonferroni post hoc test was used to determine which gaze direction positions showed significant differences in device accuracy on a black background (see Figure 8) and on a white background (see Figure 9).

**Figure 10:**
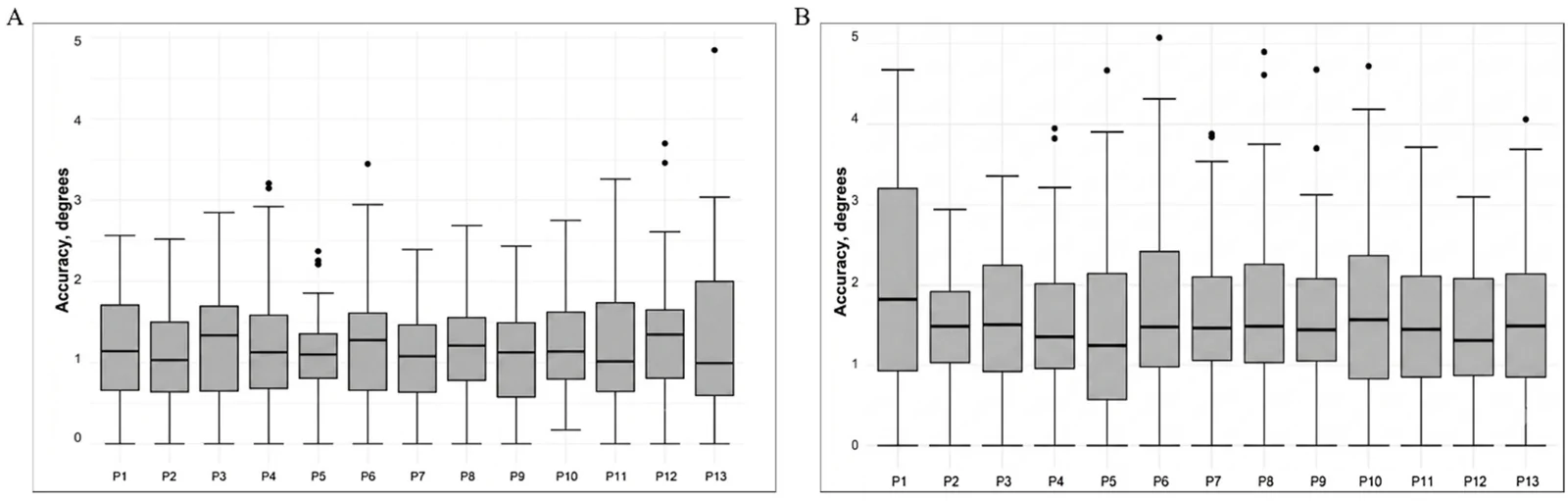
Data deviation from the stimulus location obtained with Pupil Labs Neon on a white background (**A**) and on a black background (**B**). The horizontal line within each box represents the median value.

In contrast, when analyzing how accuracy varies with stimulus position using the Pupil Labs Neon video-oculograph, it was found that the accuracy of this device does not depend on the position of the fixation stimulus on either a white (χ^2^ = 4.80; *p* = 0.964) or black (χ^2^ = 13.89; *p* = 0.308) background, as determined by the Friedman test (see Figure 10).

#### 3.1.3. Effect of Stimulus Background Color on Eye-Tracking Device Accuracy

Comparing the accuracy of the Tobii Pro Fusion video-oculograph on a white background (1.35° ± 0.85°) and a black background (1.10° ± 0.70°), as well as the accuracy of the Pupil Labs Neon video-oculograph on a white background (1.20° ± 0.72°) and a black background (1.63° ± 1.05°), the Wilcoxon signed-rank test showed that background color has a statistically significant effect on data accuracy for both the Tobii Pro Fusion device (Z = −3.512; *p* < 0.001) and the Pupil Labs Neon device (Z = −4.092; *p* < 0.001).

### 3.2. Fixation Stability

The gaze fixation stability between Tobii Pro Fusion and Pupil Labs Neon video-oculographs was compared.

**Figure 11:**
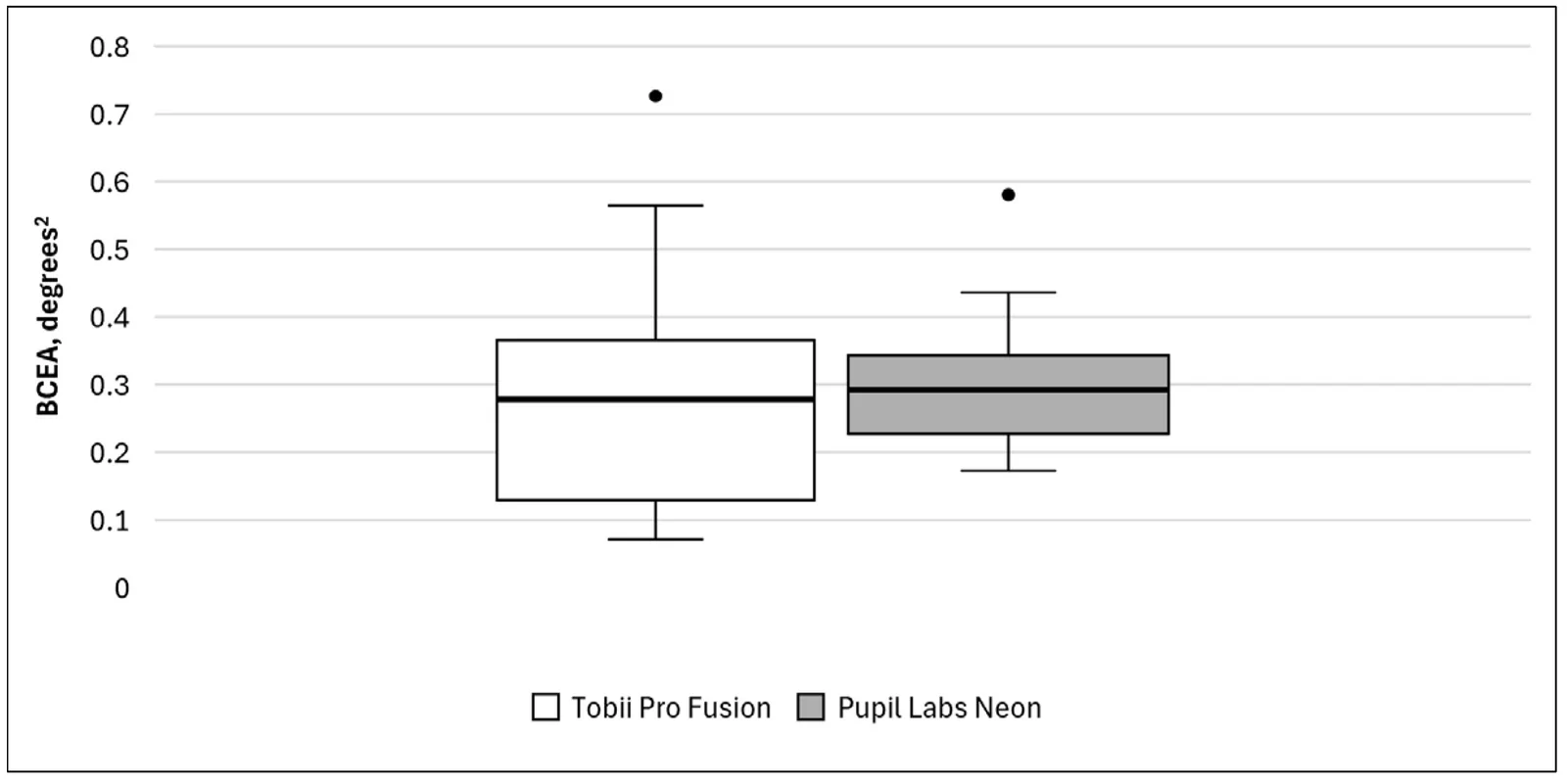
Comparison of gaze fixation stability assessed for a centrally located fixation stimulus between Tobii Pro Fusion and Pupil Labs Neon eye-tracking devices. The horizontal line within each box represents the median value.

The mean gaze fixation stability assessed with the Tobii Pro Fusion indicated slightly more stable fixation (0.28 ± 0.16 deg^2^) compared with the Pupil Labs Neon eye-tracking device (0.30 ± 0.09 deg^2^). Since the data were not normally distributed according to the Shapiro–Wilk test (*p* < 0.05), statistical comparisons were performed using the Wilcoxon signed-rank test. The difference between the gaze fixation stability values obtained with the Tobii Pro Fusion (Mdn = 0.28) and Pupil Labs Neon (Mdn = 0.29) was not statistically significant (Z = −0.833, *p* = 0.405) (see Figure 11).

## 4. Discussion

While eye-tracking technology has become widely accessible, particularly with the rise of wearable glasses that facilitate tracking outside of laboratory environments [24], it remains critical to evaluate whether these mobile systems introduce distinct limitations that could compromise data quality and interpretability.

Eye-tracking system accuracy is an essential factor in both academic research, and commercial and clinical applications [13]; therefore, the accuracy of two video-oculography devices (Tobii Pro Fusion and Pupil Labs Neon) was compared by analyzing the effects of both fixation stimulus position and background color. Although the operating frequencies of the two devices differ, according to the study by Serpa et al. [25], different sampling frequencies do not have a significant impact on fixation stability measurements. The mean accuracy obtained with the Tobii Pro Fusion eye-tracking device (1.23° ± 0.79°) was significantly higher than that obtained with the Pupil Labs Neon device (1.41° ± 0.92°). The obtained accuracy of the Tobii Pro Fusion device differed noticeably from the manufacturer-defined value (0.3° under optimal conditions). Optimal conditions are defined by the manufacturer as a room lighting of approximately 300 lux, uniform illumination without flickering and limited exposure to sunlight, no nearby devices that may emit infrared radiation, and the participants cannot have pronounced eye makeup, extremely small or large pupils, excessive tearing, prior eye surgery, and cannot have cataracts, amblyopia, strabismus, nystagmus, or ptosis. In contrast, the accuracy evaluated for the Pupil Labs Neon device is much closer to that specified in the device manual (1.8° without calibration, 1.3° with gaze offset correction) [21,26]. This observation is also consistent with the accuracy values specified by the manufacturers, as the nominal accuracy of Pupil Labs Neon is lower than that reported for Tobii Pro Fusion. This result also confirms findings reported in previous studies, indicating that in real-world conditions device accuracy may fall within the range of 1–2°, although manufacturers typically specify higher accuracy, around 0.5° [9,10,11,12].

The background color of the fixation stimulus also plays an important role in determining gaze accuracy, as the obtained device accuracy varies between background colors. The Tobii Pro Fusion device demonstrated statistically significantly higher accuracy on a dark background (1.10° ± 0.70°) compared to a light background (1.35° ± 0.85°). In contrast, the Pupil Labs Neon device showed the opposite trend—higher accuracy was observed on a light background (1.20° ± 0.72°) than on a dark background (1.63° ± 1.05°). These differences are most likely explained by differences in the pupil detection and gaze estimation algorithms of the two devices, as well as their sensitivity to changes in illumination [27]. The obtained results may also have been influenced by the calibration procedure, as calibration was performed using only a black stimulus on a white background. To maximize measurement accuracy, the calibration procedure should match the visual conditions of the experimental task [28].

Analysis of stimulus position indicated that the gaze accuracy of Tobii Pro Fusion changed significantly depending on stimulus position on the screen. This finding may reflect limitations in gaze estimation at larger viewing angles or the influence of optical distortions in peripheral regions [29]. Similar observations have been reported in previous studies evaluating the accuracy of screen-based eye trackers [9,30], which demonstrated that gaze estimates are generally most accurate near the center of the screen and become progressively less accurate toward the periphery. To compensate for this decrease in accuracy, Padikal et al. [30] proposed a mathematical correction applied after calibration, showing that systematic gaze estimation errors can often be reduced using simple linear transformations derived from multiple calibration points. In contrast, Pupil Labs Neon, whose operation is based on deep neural network models, demonstrated considerably more uniform accuracy across the screen, indicating more stable performance, highlighting the potential of machine learning in the development of eye-tracking technologies, and demonstrating the ability of deep neural network models to compensate for changes in gaze angle and geometric distortions across the entire screen area [8,21]. However, similar behaviour has not been observed for all wearable eye trackers. For example, studies evaluating Tobii Glasses 2 and Tobii Glasses 3 have reported higher accuracies when participants fixate on centrally positioned stimuli than on stimuli presented in more peripheral gaze directions [31]. In the present study, a chin and forehead rest was used during recordings with both Tobii Pro Fusion and Pupil Labs Neon to minimize head movements. Onkhar et al. [31] found that the accuracy of wearable eye trackers may improve when a chin rest is not used, as they allow natural head movements during the task. Under these conditions, gaze shifts toward peripheral targets require smaller eye rotations because part of the movement is achieved through head rotation. Therefore, although Pupil Labs Neon is a wearable eye tracker, the findings of the present study should not be generalized to all wearable eye trackers or to all experimental setups. The relatively uniform accuracy of Pupil Labs Neon across different gaze directions may be particularly important when designing experiments involving widely distributed visual stimuli or when applying eye tracking in real-world environments, where gaze direction changes dynamically and is less predictable.

Relatively few comparative studies have been conducted on eye-tracking devices’ abilities to evaluate gaze fixation stability. Liu et al. [32] compared fixation stability measurements obtained using two microperimetry devices incorporating eye-tracking technology and found no significant differences between them. In contrast, Zafar et al. [33] compared two wearable eye trackers based on different gaze estimation principles and reported that fixation stability estimates may vary between devices. In the present study, comparison of fixation stability measurements obtained with the screen-based eye tracker Tobii Pro Fusion and the wearable eye tracker Pupil Labs Neon showed that both devices provided comparable estimates of fixation stability within the central area of the screen. Although fixation stability measurements obtained with Tobii Pro Fusion exhibited greater variability, potentially reflecting its higher sensitivity to small fixation eye movements [5], no significant differences in fixation stability estimates were observed between the two devices.

Although eye-tracking accuracy was evaluated at 13 stimulus positions spanning both central and peripheral regions of the screen, fixation stability was analysed only during central fixation. Similar approaches to the quantitative analysis of fixation stability have been adopted in previous studies [33,34,35,36].

It should be noted that during data processing, data loss was observed in recordings obtained with Pupil Labs Neon at all fixation target positions, including central fixation. However, a detailed analysis of the amount of data loss and the factors contributing to it was beyond the scope of the present study. Nevertheless, several factors reported in the literature may potentially explain the occurrence of missing gaze samples. Each eye-tracking system utilizes specialized software pipelines to translate ocular images and specific parameters into continuous gaze coordinates. The performance of these algorithms often depends on the quality of the captured pupil video signal [37]. If the algorithm is unable to reliably identify the necessary ocular or pupil features, gaze samples may not be recorded. Comparisons of pupil detection algorithms used in head-mounted eye trackers have shown that the algorithm implemented in Pupil Labs devices has one of the worst pupil detection performances among the evaluated methods [38]. It should be noted that this study refers to the algorithm used in the Pupil Labs Core device; since the newer Pupil Labs Neon device uses an improved algorithm based on deep learning [3], further studies are required to compare these algorithms.

## 5. Conclusions

The results of the present study demonstrated that the overall accuracy of the Tobii Pro Fusion eye-tracking device was significantly higher than that of the wearable Pupil Labs Neon device. However, Tobii Pro Fusion’s accuracy varied with increasing gaze eccentricity, whereas Pupil Labs Neon exhibited relatively uniform accuracy across different gaze directions. The stimulus background color also influenced the accuracy of both devices, with Tobii Pro Fusion performing better on a dark background and Pupil Labs Neon on a light background.

No significant differences in fixation stability estimates during central fixation were observed between Tobii Pro Fusion and Pupil Labs Neon.

Overall, Tobii Pro Fusion appears to be better suited for screen-based gaze fixation analysis, whereas Pupil Labs Neon may be suitable for applications where relatively uniform accuracy across different gaze directions is advantageous. Future studies should further evaluate the performance of Pupil Labs Neon under different experimental conditions and investigate data quality metrics, including the extent of data loss, in greater detail.

## Data Availability

The data presented in this study are available on request from the corresponding authors. The data are not publicly available due to privacy reasons.
